# Assessment of disinfectant efficacy in reducing microbial growth

**DOI:** 10.1371/journal.pone.0269850

**Published:** 2022-06-27

**Authors:** Abdullah A. Alajlan, Lenah E. Mukhtar, Adnan S. Almussallam, Abdullah M. Alnuqaydan, Nasser S. Albakiri, Turki F. Almutari, Khalid M. Bin Shehail, Fahad S. Aldawsari, Sulaiman M. Alajel

**Affiliations:** 1 Microbial Identification Division, Reference Laboratory for Microbiology, Executive Department of Reference Laboratories, Research and Laboratories Sector, Saudi Food and Drug Authority (SFDA), Riyadh, Saudi Arabia; 2 Antimicrobial Resistance Division, Reference Laboratory for Microbiology, Executive Department of Reference Laboratories, Research and Laboratories Sector, Saudi Food and Drug Authority (SFDA), Riyadh, Saudi Arabia; 3 Drug and Cosmetic Reference Laboratory, Executive Department of Reference Laboratories, Research and Laboratories Sector, Saudi Food and Drug Authority (SFDA), Riyadh, Saudi Arabia; 4 Department of Medical Biotechnology, College of Applied Medical Sciences, Qassim University, Buraidah, Saudi Arabia; 5 Drug and Cosmetic Control Laboratory, Executive Department of Laboratory, Research and Laboratories Sector, Saudi Food and Drug Authority (SFDA), Riyadh, Saudi Arabia; 6 Reference Laboratory for Microbiology, Executive Department of Reference Laboratories, Research and Laboratories Sector, Saudi Food and Drug Authority (SFDA), Riyadh, Saudi Arabia; Hodeidah University, YEMEN

## Abstract

The incidence of hospital- and community-acquired infections has been dramatically increased worldwide. Accordingly, hands hygiene and the use of disinfectants have been increased leading to the expansion in hand sanitizers production to meet public demand. This study was conducted to assess the efficiency of common disinfectants in the market of Riyadh, Saudi Arabia in inhibiting the microbial growth during the time of Coronavirus disease 2019 (COVID-19) pandemic. Five bacterial strains of commonly hospital-acquired infections (*Pseudomonas aeruginosa*, *Escherichia coli*, *Salmonella enteritidis*, *Staphylococcus aureus*, and *Enterococcus faecalis*) (ATCC reference strains and clinical isolates) were examined for their susceptibility against 18 disinfectants collected from the Saudi market. The tested 18 disinfectants were broadly clustered into different groups based on their active chemical composition as following: 12 products contained alcohol, 2 products had chlorhexidine, 3 products contained mixed concentration of alcohol/chlorhexidine and 1 product had a mixture of chlorhexidine/Hexamidine/Chlorocresol. By measuring the minimum inhibitory concentration (MIC) and the minimum bactericidal concentration (MBC), our results revealed that all the 18 disinfectants have reduced the microbial growth of all the tested strains. Generally, the MICs and the MBCs for the clinical strains are higher than those of the reference strains. Taken together, our findings showed that all tested products have high disinfectants’ killing rate against microbes of different origins, which suggest the high quality of these disinfectants and the good surveillance practice by the local authorities in Saudi Arabia.

## Introduction

In recent years, the rate of hospital- and community-acquired infections has been increasing globally [1,2]. Hands are the most common route of transmission of these nosocomial diseases [1,3]. According to Tri-Country Healthcare 2018 report, more than 80% of human illnesses can be transmitted by hands [4]. The most common resident microbial flora habitation on the deeper skin layers of the hands are *Staphylococcus aureus* (*S*. *aureus*), *Staphylococcus epidermidis* (*S*. *epidermidis*), and *Enterococcus faecalis* (*E*. *faecalis*) [1]. On the other hand, *Escherichia coli (E*. *coli)*, *Salmonella enteritidis* (*S*. *enteritidis*) *and Pseudomonas aeruginosa* (*P*. *aeruginosa*) are common transient floras, where they temporary colonize the superficial layers of the hands [1]. The spread of these hand microbes mainly depends on the type and the density of pathogenic microorganism and patient’s immune system [1,3].

According to the Center for Disease Control and Prevention (CDC), every year, about 2 million people acquire nosocomial infections worldwide and about 90,000 deaths as a result of these infections [5,6].

The combination of the pathogenic microorganism and drug-induced immunosuppression possibly rises the vulnerability to secondary infections [7]. Secondary infection is the infection that occurs during or after treatment for a previous infection [8]. A number of retrospective studies revealed that some of the hospitalized patients developed respiratory, urinary, or blood-stream secondary infections [9]. The most commonly reported pathogens associated with these secondary infections were the Gram-negative bacteria (*Klebsiella pneumoniae*, *E*. *coli*, *P*. *aeruginosa*, and *Acinetobacter baumannii*), followed by Gram-positive bacteria (*Staphylococcus haemolyticus*, *Staphylococcus epidermidis*, *Enterococcus faecium*, *E*. *faecalis*), then viruses (HSV-1) and fungi *Candida albicans* (*C*. *albicans*) [9]. Therefore, the CDC, the World Health Organization (WHO), and the Food and Drug Administration (FDA) suggested that hand hygiene is extremely important measure to prevent or control the spread of infectious diseases caused by severe acute respiratory syndrome coronavirus 2 (SARS-CoV-2) and other hospital-acquired and community-associated infections [6,10]. Hand hygiene can be done either by hand washing or by hand sanitizing such as alcohol-based products [2,5,11,12].

Hand sanitizers have been reported as a convenient and fast way to decontaminate and eliminate the growth of the pathogenic microbes present on an individuals’ hands in the absence of water and soap [2,5,11]. Hand sanitizers can be divided into 2 classes according to their active ingredients: (i) alcohol-based hand sanitizer, and (ii) non-alcohol-based hand sanitizer. The efficacy of hand sanitizers (ethanol or isopropanol) depends on several factors such as (i) the type of alcohol, (ii) the concentration of the active ingredients, (iii) the exposure time, and (iv) the amount of used disinfectant [13].

Alcohol-based hand rub (ABHR) sanitizers are the most reported hand sanitizer this far, which are often comprised of ethanol or isopropanol alcohol [2,5,11,12]. The FDA, CDC, and WHO recommend a concentration of 60% to 95% ethanol or isopropanol in alcohol-based hand sanitizers products to reduce microbial burden by causing cellular proteins denaturation, lipids dissolution, and disruption of tissue membranes of microbes and inactivate viral functions [1,2,5,11,12,14]. However, the frequent use of these alcoholic sanitizers can lead to skin dryness unless emollient is added to the formulation to helps in skin hydration [15]. Glycerin is the most common emollient added to alcohol-based sanitizers [15,16]. Therefore, the FDA also recommends that ABHR sanitizers should contain 1.45% glycerin (a gel-forming substance) to maintains hand moisturization and prevents skin dryness from the alcohol effect [15,16]. Several studies have demonstrated the antimicrobial activity of ABHR and its efficacy in eliminating the microbial growth of clinical strains of Gram-positive and Gram-negative bacterial pathogens including *S*. *aureus*, *E*. *faecalis*, *E*. *coli*, *P*. *aeruginosa*, *Acinetobacter baumannii* (*A*. *baumannii*), and *C*. *albicans* of human volunteers [2,11,16–19]. ABHR also have proven to deliver antiviral effect against both enveloped and nonenveloped viruses of immediate health concern such as: influenza A virus, Middle Eastern respiratory syndrome (MERS) virus, Zika virus, Ebola virus, severe acute respiratory syndrome coronavirus (SARSCoV), and the newly emerged SARS-CoV-2 [11,20–23]. Where in fact, 70% ethanol with 1.45% glycerin was the most effective concentration reported this far and the preferable by health workers [1,24,25]. However, there are some limitations of these alcohol-based disinfectants, including flammable susceptibility, skin toxicity in case of high-alcohol concentration, and short half-life as an antimicrobial agent.

On the other side, chlorohexidine, is a non-alcohol-based disinfectant. It has antiseptic, pharmaceutical, and antiplaque activities against different microbes [24,26–28]. Chlorohexidine has shown to act as bactericidal agent against both Gram-positive and Gram-negative bacteria by increasing microbial cell membrane permeability, altering protein functions which leading to the precipitation of macromolecules in the cytoplasm and subsequently microbial cell death [26,27,29]. It has also bacteriostatic activity due to the inactivation of ATPases preventing prokaryotic cells replication [26,27,29].

COVID-19 pandemic has brought a shortage of hand sanitizers all around the world [30]. In response to this pandemic, there has been increased in the awareness and the need of using hand sanitizers. To meet this need, the Saudi Food and Drug Authority (SFDA) has released some temporarily relaxed guidance to manufacturers regarding the production of hand sanitizers. As a result, many companies have launched many brands of hand sanitizer in the market without verifying the proper concentration of their ingredients nor the activity of these agents against microbes [5,12]. In fact, these companies claim in their hand sanitizers labels that these products are effective and have a microbial killing efficiency by 99.9% [5,12,31]. Thus, it is important to test the efficacy of these disinfectant products in reducing growth of food- and hospital-bacterial isolates. Most importantly, it is necessary to verify these claims by regulatory authorities to enforce top-quality hygiene measures for the prevention of microbial infections. To overcome this ambiguity, this study was conducted to evaluate the antimicrobial activity of the common brands of disinfectant available in the Saudi market against both reference ATCC strains and clinical isolates of *P*. *aeruginosa*, *E*. *coli*, *S*. *enteritidis*, *S*. *aureus*, and *E*. *faecalis*.

## Materials and methods

### Bacterial reference strains

Five reference strains *Pseudomonas aeruginosa* (ATCC 27853), *Escherichia coli* (ATCC 25922), *Salmonella enterica* subsp. *enterica* (ATCC 13076), *Staphylococcus aureus* (ATCC 25923), and *Enterococcus faecalis* (ATCC 19433) were used in this study. These reference strains were chosen based on their availability in the SFDA’s laboratory. During the study, each strain was inoculated on its own selective media and incubated overnight at 37°C for colony isolation. Next, Gram stain test was performed to differentiate between Gram-positive and Gram-negative-bacteria. After that, biochemical tests were done using Matrix-Assisted Laser Desorption/Ionization-Time of Flight mass spectrometry (MALDI-TOF MS) to confirm the identity of the bacterial species strain. Bacterial strains were cultured on tryptic soy agar (TSA) to examine the efficacy of different disinfectant products.

### Bacterial clinical isolates

The clinical isolates of *P*. *aeruginosa*, *E*. *coli*, *Salmonella enteritidis*, methicillin-resistant *S*. *aureus* (MRSA), and *E*. *faecalis* were used in the study to test the effectiveness of different disinfectant products. These clinical stains were isolated from hospitalized patients and were obtained from Dr. Majed Alghoribi (Infectious Disease Research Department, King Abdullah International Medical Research Center’s (KAIMRC)). The isolates were identified using VITEK 2 system (bioMérieux) and at the molecular level by polymerase chain reaction (PCR) targeting a species-specific fragment of the 16S rRNA gene for all the organisms and *oprL* gene for *P*. *aeruginosa* at KIMARC. Then, the identification of these isolates was confirmed at our facility by MALDI-TOF MS technology, except the clinical *E*. *coli isolate* as it was confirmed at the species level by PCR.

### Disinfectants

The ten tested bacteria (5 reference ATCC strains and 5 clinical isolates) were evaluated independently for their antimicrobial sensitivity against the 18 products of disinfectants purchased from the local market in Riyadh, Saudi Arabia. These products were selected as representative of 3 categories (i) alcohol-based (contain ethanol or isopropanol), (ii) chlorohexidine-based (contain chlorhexidine gluconate (CHG), and (iii) mixed product (combination of both alcohol and CHG active ingredients). In brief, there were 12 alcoholic products, 11 out 12 contained ethanol as an active ingredient (products 1–8, 15–16, and 18), and one product had isopropyl alcohol (product 7). Two products had chlorhexidine gluconate (products 11 and 12), three products contained mixed concentrations with different percentages of isopropyl alcohol and chlorhexidine gluconate (products 10, 13, and 14), and one product had a mixture concentration of chlorhexidine gluconate/Hexamidine disethionate/Chlorocresol (product 9). These 18 disinfectants and their active ingredients are listed in Table 1.

**Table 1:** This table lists the 18 disinfectant products collected from the local market of Riyadh, Saudi Arabia.

Hand Sanitizer Product #	Category	Concentration as labeled in the bottle	Ethanol% measured by GC-FID	Isopropyl % measured by GC-FID	Glycerin % measured by GC-MS
Product 1	Alcohol	Ethanol 70% v/v	75.4	--	0.32
Product 2	Alcohol	Ethanol 70–80% v/v	85.38	--	1.55
Product 3	Alcohol	Ethanol 70% v/v	80.94	--	1.21
Product 4	Alcohol	Ethanol 70% v/v	82.13	--	1.34
Product 5	Alcohol	Ethanol 70% v/v	87	--	1.06
Product 6	Alcohol	Ethanol 70% v/v	80	--	1.19
Product 7	Alcohol	Ethanol 70% v/v	60	--	1.07
Product 8	Alcohol	Ethanol 70% v/v	77	--	0.41
Product 9	Mixed	1% Chlorhexidine Gluconate, 1% Hexamidine, 3% Chlorocresol**	--	--	N.D*
Product 10	Mixed	4% Chlorhexidine Gluconate, 4% w/v Isopropyl Alcohol	--	4.7	0.37
Product 11	chlorohexidine	Chlorhexidine Gluconate 2%**	--	--	0.36
Product 12	chlorohexidine	Chlorhexidine Gluconate 4%**	--	--	0.33
Product 13	Mixed	Chlorhexidine Gluconate 0.5%, Isopropyl Alcohol 70% w/v	--	100	N.D
Product 14	Mixed	Chlorhexidine Gluconate 0.5%, Isopropyl Alcohol 70% w/v	--	93	N.D
Product 15	Alcohol	Ethanol 70% v/v	77	--	0.92
Product 16	Alcohol	Ethanol 70% v/v	92	--	1.66
Product 17	Alcohol	Isopropyl 70% v/v	--	91	N.D
Product 18	Alcohol	Ethanol 70–80% v/v	87.2	--	1.19

* N.D means not detected by GC-MS either no peaks found at same retention time or library results does not match glycerin spectrum.

** The label of the product has no v/v or w/v expression for active ingredient.

### Minimum inhibitory concentration (MIC) determination

Broth microdilution assay and inoculum preparation were performed on a 96-well microtitre flat bottom plate that has a lid to prevent alcohol evaporation (Catalog number: 266120, Thermo Fisher Scientific, NY, USA) and the experimental design was followed according to Schug *et al*. with slight modifications [32]. Briefly, 2-fold disinfectant serial dilutions were prepared in 100 μL sterile demineralized water. A total of 100 μl of demineralized water was added to each well of a sterile 96-well microtiter plate except for the first row (raw A) in which 100 μl of each disinfectant (from the stock bottle) was added. Then, 100 μl of demineralized water was added to the disinfectant in raw A using a multichannel pipette and mixed. Then, 100 μl of the homogenize solution was transferred to the second raw (raw B) and mixed. Then, 100 μl of the homogenize solution was transferred to the third raw (raw C) and mixed. This homogenization steps were repeated sequentially until the lowest test concentration in raw G was reached. After that, 100 μl of the mixture from raw G was discarded. Thus, this resulted in a 2-fold concentration solution in which all the rows A-G contained 100 μl of 2-fold diluted disinfectant solution. The last row H has no disinfectant and served as growth control.

Once the preparation of the disinfectant solutions was completed, bacterial inoculum suspensions were prepared. Inoculum preparation was performed according to Schug *et al*. with slight modifications [32]. Fresh bacterial colonies were inoculated on tryptic soy agar (TSA) plates and then in tryptone-saline-diluent (TSD) broth to produce a homogeneous suspension. Three serial dilution steps were performed on the homogenized bacterial suspension as sterility controls to determine the final concentration of bacteria for the MIC. Then, the optical density was measured with a spectrophotometer at 625 nm and adjusted to 0.085–0.077. At the same time, a total of 10 μl of the inoculum suspension was diluted in 10 ml TSD and then 100 μl of the suspension were plated on TSA in duplicates. Then, after 24 h incubation at 37°C, colonies were counted and calculation to confirm the concentration of the inoculum suspension was within 1 x 108–1 x 10^9^ CFU/ml, which is equivalent to the 0.5 McFarland turbidity standard [32].

After that, vigorous vortexing the diluted bacterial suspension was performed, then by using a multichannel, 100 μl of the bacterial suspension was transferred into each well of the microtiter plate resulting in a final volume of 200 μl/well and a concentration of 1x TSB. In addition, 2 wells from the 96-wells plate were used for positive growth control (100 μl of each bacterial inoculum mixed with 100 μl sterile water) and negative growth control (100 μl TSB 2x mixed with 100 μl sterile water). Then, all the plates were incubated at 37°C for 24 h. MIC was defined as the lowest concentration of the disinfectant that does not yield visible bacterial growth following 24 h incubation.

### Minimum bactericidal concentration (MBC)

MBC testing was carried out to determine the bactericidal effect of each disinfectant that kill the growth of bacteria. In brief, 10 μl from each well of the microtitre plate (MIC assay) that showed visible, and no visible growth were further spotted on TSA agar and incubated at 37°C for 24 h and were observed for growth. The lowest concentration that does not show any bacterial growth was determined as the MBC.

### Determination of glycerin in disinfectant products using gas chromatography—mass spectrometry (GC-MS)

Analysis of glycerin in the tested disinfectants was performed using an Agilent 7890 / 5975C GC-MS equipped with a DB-5MS capillary column (30 m x 0.25 mm x 0.25 μm) containing ultra-inert nonpolar (5%-phenyl)-methylpolysiloxane column as a stationary phase with helium as the carrier gas. Glycerin quality control standards (0.00250–0.02500% v/v) were prepared by diluting glycerin stock (99.9%, Merck) in methanol HPLC grade (Merck). Samples were diluted 100X using methanol HPLC grade followed by 5 minutes shaking of the solution. The column was coupled to a mass spectrometer for mass detection of fragments ranges between 25–450 *m/z*. These standards were used in between samples to ensure the accuracy of the sampling results. GC-MS method was adapted to quantify glycerin using two-dimensional confirmations, by standard and by mass spectrum using NIST library, to match standards results to the tested samples, and to determine the accuracy of the results. Standards and samples were identified by the retention time and calculated as area ratio between standards and sampless.

### Determination of alcohol in disinfectant products using gas chromatography—flame ionization detector (GC-FID)

The identification and the quantification of alcohol in the tested disinfectants was performed using GC- flame ionization detector (GC-FID) through liquid injection technique by Agilent 7890A equipped with a phase G43 capillary column (0.32 mm × 30 m x1.8 μm). Separation performed by Intermediate-Polar (6% Cyanopropylphenyl / 94% dimethyl polysiloxane column as stationary phase. Alcohol quality control standards (1.0–7.0% v/v) were prepared by diluting alcohol stock in deionized water. Samples were diluted 50X using deionized water followed by 5 minutes shaking of the solution. Standards and samples were identified by the retention time and calculated as area ratio between standards and samples.

### Data analysis

The presented data were analyzed and stored in Excel Microsoft spreadsheet. The error bars represent the mean ± standard deviation (SD) of 3 independent biological experimental replicates (n = 3). Statistical significances for MICs of alcohol and glycerin correlation were analyzed using GraphPad Prism software version 9.0 (GraphPad, La Jolla, CA, USA) and determined using Welch’s t-test with a 95% confidence interval for comparing the means between 2 independent groups without assuming equal population variances. The *p*-value equal to or less than ≤ 0.05 is considered statistically significant.

## Results

### Identification of disinfectants active ingredients

The selected 18 disinfectants were analyzed using GC-MS and GC-FID methodologies to provide a full assessment of their alcohol and glycerin contents (Table 1). Eleven products (products 1–8, 15–16, and 18) contained varying percentages of ethanol and glycerin; where products 2–6 and 18 contained more than 80% alcohol supplemented with more than 1% glycerin (ranges between 1.06%– 1.55%). Product 7 contained 1.07% glycerin and 60% ethanol. In the other alcohol-based disinfectants (products 1, 8, and 15), ethanol concentrations were 75.4%, 77%, and 77%, whereas glycerin contents were 0.32%, 0.41%, and 0.92%, respectively. However, glycerin concentration did not exceed the 1.45%, except in product 2 and 16. One product (product 17) contained isopropyl alcohol, while glycerin concentration was not detected. The results of the ethanol, isopropyl and glycerin concentrations are shown in Table 1.

### Antimicrobial effect of disinfectant products against ATCC reference strains

The goal of this study was to assess the antibacterial activities (inhibitory and bactericidal effects) of the 18-commercial disinfectants from different forms (gels, sprays, and solutions) against 5 ATCC reference and 5 clinical isolates bacteria. Overall, our results demonstrated that all the tested disinfectant products reduced and inhibited microbial growth at or below the qualified concentration of the active ingredient (Tables 2 and 3, S1 and S2 Figs). Bacterial growth of the selected test 5 organisms was counted in colony-forming unit across MBC concentration of the 18 disinfectants (Table 3). Alcohol-based disinfectants presented good efficacy in inhibiting bacterial growth against both ATCC reference strains and clinical isolate strains. However, the rate of the inhibition was lower than the chlorohexidine-based and mixed products. Interestingly, disinfectant products contained chlorhexidine and mixture of chlorhexidine/isopropyl alcohol showed promising antimicrobial effects against all the strains used in this study (Tables 2 and 3).

**Table 2:** This table demonstrates the MIC% of all disinfectant products in inhibiting the growth of five bacterial species of ATCC reference strain and clinical isolates*.

Hand Sanitizer Product #	MIC (%) of Tested Sanitizers Against Reference Strains Gram-negative Gram-positive					MIC (%) of Tested Sanitizers Against Clinical Isolates Gram-negative Gram-positive				
	*P*. *aeruginosa*	*E*. *coli*	*S*. *enteritidis*	*S*. *aureus*	*E*. *faecalis*	*P*. *aeruginosa*	*E*. *coli*	*S*. *enteritidis*	*S*. *aureus*	*E*. *Faecalis*
**Product 1**	25.00	25.00	25.00	50.00	75.00	25.00	25.00	50.00	50. 00	75.00
**Product 2**	25.00	50.00	50.00	75.00	75.00	25.00	50.00	50.00	75.00	75.00
**Product 3**	75.00	50.00	50.00	50.00	75.00	25.00	50.00	50.00	75.00	75.00
**Product 4**	50.00	25.00	25.00	50.00	100.00	25.00	50.00	25.00	50.00	75.00
**Product 5**	25.00	50.00	50.00	75.00	100.00	50.00	50.00	50.00	75.00	75.00
**Product 6**	25.00	50.00	50.00	75.00	75.00	50.00	50.00	50.00	75.00	75.00
**Product 7**	50.00	50.00	50.00	75.00	75.00	50.00	50.00	50.00	75.00	75.00
**Product 8**	25.00	50.00	50.00	75.00	75.00	50.00	50.00	50.00	100.00	75.00
**Product 9**	50.00	25.00	25.00	50.00	50.00	50.00	50.00	25.00	12.50	50.00
**Product 10**	0.78	0.78	0.78	0.78	0.78	0.78	0.78	0.78	0.78	0.78
**Product 11**	0.78	0.78	0.78	0.78	0.78	0.78	0.78	0.78	0.78	0.78
**Product 12**	0.78	0.78	0.78	0.78	0.78	0.78	0.78	0.78	0.78	0.78
**Product 13**	1.56	0.78	0.78	0.78	1.56	0.78	0.78	0.78	0.78	1.56
**Product 14**	0.78	0.78	0.78	0.78	1.56	0.78	0.78	0.78	0.78	0.78
**Product 15**	25.00	25.00	50.00	75.00	75.00	25.00	25.00	50.00	100.00	75.00
**Product 16**	50.00	50.00	50.00	75.00	75.00	50.00	50.00	25.00	100.00	75.00
**Product 17**	25.00	25.00	50.00	50.00	75.00	25.00	25.00	25.00	100.00	75.00
**Product 18**	50.00	50.00	50.00	75.00	75.00	25.00	50.00	50.00	100.00	75.00

*The experiments were performed 3 times and one reproducible data has given.

**Table 3:** This table demonstrates the MBC % and total number of bacterial colony of all isolates towards all disinfectant products *.

HAND SANITIZER PRODUCT #	MBC % & CFU/ML	REFERENCE STRAINS					CLINICAL ISOLATES				
		Gram-negative			Gram-positive		Gram-negative			Gram-positive	
		*P*. *aeruginosa*	*E*. *coli*	*S*. *enteritidis*	*S*. *aureus*	*E*. *faecalis*	*P*. *aeruginosa*	*E*. *coli*	*S*. *enteritidis*	*S*. *aureus*	*E*. *faecalis*
**PRODUCT 1**	MBC%	12.50	12.50	12.50	25.00	50.00	12.50	12.50	25.00	25.00	50.00
	CFU/ml	74.00	55.00	TMCC	300.00	56.00	65.00	42.00	TMCC	77.00	50.00
**PRODUCT 2**	MBC%	12.50	25.00	25.00	50.00	50.00	12.50	25.00	25.00	50.00	50.00
	CFU/ml	81.00	53.00	120.00	TMCC**	53.00	71.00	80.00	250.00	85.00	46.00
**PRODUCT 3**	MBC%	50.00	25.00	25.00	25.00	50.00	12.50	25.00	25.00	50.00	50.00
	CFU/ml	TMCC	43.00	100.00	280.00	TMCC	TMCC	43.00	260.00	TMCC	TMCC
**PRODUCT 4**	MBC%	25.00	12.50	12.50	25.00	75.00	12.50	25.00	12.50	25.00	50.00
	CFU/ml	400.00	TMCC	TMCC	TMCC	TMCC	TMCC	16.00	TMCC	TMCC	TMCC
**PRODUCT 5**	MBC%	12.50	25.00	25.00	50.00	75.00	25.00	25.00	25.00	50.00	50.00
	CFU/ml	TMCC	290.00	63.00	TMCC	TMCC	TMCC	TMCC	280.00	TMCC	TMCC
**PRODUCT 6**	MBC%	12.50	25.00	25.00	50.00	50.00	25.00	25.00	25.00	50.00	50.00
	CFU/ml	TMCC	TMCC	TMCC	TMCC	TMCC	250.00	TMCC	200.00	TMCC	TMCC
**PRODUCT 7**	MBC%	25.00	25.00	25.00	50.00	50.00	25.00	25.00	25.00	50.00	50.00
	CFU/ml	350.00	TMCC	TMCC	TMCC	TMCC	TMCC	260.00	300.00	TMCC	TMCC
**PRODUCT 8**	MBC%	12.50	25.00	25.00	50.00	50.00	25.00	25.00	25.00	75.00	50.00
	CFU/ml	TMCC	TMCC	TMCC	TMCC	TMCC	200.00	TMCC	180.00	158.00	TMCC
**PRODUCT 9**	MBC%	25.00	12.50	12.50	25.00	25.00	25.00	25.00	12.50	6.25	25.00
	CFU/ml	TMCC	TMCC	TMCC	TMCC	TMCC	TMCC	TMCC	TMCC	TMCC	41.00
**PRODUCT 10**	MBC%	No***	No	No	No	No	No	No	No	No	No
	CFU/ml	--	--	--	--	--	--	--	--	--	--
**PRODUCT 11**	MBC%	No	No	No	No	No	No	No	No	No	No
	CFU/ml	--	--	--	--	--	--	--	--	--	--
**PRODUCT 12**	MBC%	No	No	No	No	No	No	No	No	No	No
	CFU/ml	--	--	--	--	--	--	--	--	--	--
**PRODUCT 13**	MBC%	0.78	No	No	No	0.78	No	No	No	No	0.78
	CFU/ml	--	--	--	--	47.00	--	--	--	--	200.00
**PRODUCT 14**	MBC%	No	No	No	No	0.78	No	No	No	No	No
	CFU/ml	--	--	--	--	--	--	--	--	--	--
**PRODUCT 15**	MBC%	12.50	12.50	25.00	50.00	50.00	12.50	12.50	25.00	75.00	50.00
	CFU/ml	TMCC	TMCC	TMCC	TMCC	5.00	TMCC	TMCC	TMCC	TMCC	TMCC
**PRODUCT 16**	MBC%	25.00	25.00	25.00	50.00	50.00	25.00	25.00	12.50	75.00	50.00
	CFU/ml	TMCC	TMCC	250.00	TMCC	TMCC	TMCC	TMCC	48.00	TMCC	TMCC
**PRODUCT 17**	MBC%	12.50	12.50	25.00	25.00	50.00	12.50	12.50	12.50	75.00	50.00
	CFU/ml	150.00	TMCC	200.00	TMCC	TMCC	84.00	TMCC	TMCC	TMCC	TMCC
**PRODUCT 18**	MBC%	25.00	25.00	25.00	50.00	50.00	12.50	25.00	25.00	75.00	50.00
	CFU/ml	TMCC	TMCC	TMCC	TMCC	TMCC	TMCC	24.00	TMCC	83.00	TMCC

* All the experiments were performed 3 times and one reproducible data has given.

** TMCC, too many colony-counts.

*** No, there was no MBC value of the tested serial dilutions.

—no CFU/ml was obtained.

Our results showed that *P*. *aeruginosa* and *E*. *coli* were the most sensitive organisms to all the 18 products compared to the other tested bacterial strains in this study (Tables 2 and 3, S3 Fig). In *P*. *aeruginosa*, seven out of 12 alcohol-based disinfectants (products 1, 2, 5, 6, 8, 15, and 17) exhibited MIC and MBC values of 25% and 12.5% of the original stock, respectively. The others (products 4, 7, 16, and 18) showed MIC and MBC values of 50% and 25% of the original stock, respectively, while product 3 had MIC concentration of 75% and MBC concentration of 50% of the original stock.

*E*. *coli* is the second sensitive organism to all disinfectants tested in this study. Four out of 12 alcohol-based products (products 1, 4, 15, and 17) exhibited MIC and MBC values of 25% and 12.5% of the original stock, respectively, while the other products (products 2, 3, 5–8, 16, and 18) showed MIC and MBC values of 50% and 25% of the original stock, respectively, against *E*. *coli*.

The MIC and MBC values of *S*. *enteritidis* toward alcohol-based products were 50% and 25% of the original stock, respectively, for all the products (products 2, 3, and 5–18), except products 1 and 4, where they had MIC and MBC values of 25% and 12.5% of the original stock, respectively (Tables 2 and 3, S5 Fig).

*S*. *aureus* and *E*. *faecalis* were more tolerant to alcohol-based products in comparison with the other organisms of this study (Tables 2 and 3, S6 and S7 Figs). *S*. *aureus* presented MIC and MBC values of 75% and 50% of the original stock, respectively, towards alcohol-based products, except four products (products 1, 3, 4 and 17), which showed MIC and MBC values at 50% and 25% of the original stock, respectively. *E*. *faecalis* seemed to be even more tolerant towards alcohol-based products compared to *S*. *aureus*. *E*. *faecalis* had MIC and MBC values of 75% and 50% of the original stock, respectively, for all products except two (products 4 and 5), where these 2 products showed higher MIC and MBC (about 100% and 75% of the original stock, respectively).

Interestingly, all the 5 reference strains exhibited MIC and MBC values of 25–50% and 12.5–25% of the original stock towards the mixed disinfectants (product 9), while the chlorohexidine-based disinfectants (products 10 to 14) showed MIC value at 0.78–1.56% of the original stock, without MBC value of the tested serial dilutions (Tables 2 and 3, S3–S7 Figs).

Next, the significance correlation between glycerin concentration and ethanol-antimicrobial efficacy was tested against the 5 reference strains using the welch’s t-test. The mean of all the alcohol-based products that have higher glycerin content (i.e., higher than the WHO recommended concentration, >1.45%) were compiled and compared to the mean of all alcohol-based products that have lower glycerin content (i.e., lower than the WHO recommended concentration, <1.45%). Throughout the analysis, it was found that the antimicrobial effect of the alcoholic-based disinfectants is almost comparable against the tested organisms regardless the glycerin concentration (*p* > 0.05, Table 4).

**Table 4:** This table demonstrates the correlation between glycerin content and the MIC of alcohol-based disinfectants against five bacterial species of ATCC reference strain and clinical isolates.

	Organism	Glycerine > 1.45%	Glycerine < 1.45%	*p*-value	Summary
		MIC % (Mean)*	MIC % (Mean)*		
**Reference Strains**	*P*. *aeruginosa*	37.5	38.88	0.7896	ns**
	*E*. *coli*	50	41.66		
	*S*. *enteritidis*	75	44.44		
	*S*. *aureus*	75	66.66		
	*E*. *faecalis*	75	80.55		
**Clinical Isolates**	*P*. *aeruginosa*	37.5	36.11	0.9189	ns
	*E*. *coli*	50	44.44		
	*S*. *enteritidis*	37.5	47.22		
	*S*. *aureus*	87.5	77.77		
	*E*. *faecalis*	75	75		

* MIC % (mean) represents the mean for all the disinfectant products that have a glycerine concentration above or below the recommended concentration by the WHO (1.45%).

** ns, non-significant.

### Antimicrobial effect of disinfectant products against clinical isolates

*P*. *aeruginosa*, *E*. *coli* and *S*. *enteritidis* seem to be susceptible to ethanol-based products (Tables 2 and 3, S3–S5 Figs). *P*. *aeruginosa* were more sensitive to all products involve in this study comparing to the other clinical isolates. In *P*. *aeruginosa*, seven out of 12 alcohol-based disinfectants (products 1–4, 15, 17, and 18) exhibited MIC and MBC values of 25% and 12.5% of the original stock, respectively. The rest of the 5 disinfectants (products 5–8 and 16) showed MIC and MBC values of 50% and 25% of the original stock, respectively.

*E*. *coli* presented MIC and MBC values of 50% and 25% of the original stock, respectively, in the alcohol-based disinfectants (products 2–8, 16, 18). Likewise, *E*. *coli* had MIC and MBC values of 25% and 12.5% of the original stock, respectively, in the alcohol-based disinfectants (products 1, 15 and 17).

In the case of *S*. *enteritidis*, the MIC and MBC were recorded at 50% and 25% of the original stock, respectively, in 9 out 12 of alcohol-based disinfectants (products 1–3, 5–8, 15, and 18). On the other hand, products 4, 16, and 17 of these alcohol-based disinfectants had 25% MIC and 12.5% MBC of the original stock.

The MIC and MBC for all the tested disinfectant products against bacteria obtained from clinical isolates demonstrated that the Gram-positive (*S*. *aureus*) and the Gram-negative (E. *faecalis*) were the most tolerant bacteria to all the 18 disinfectants in contrast with the other clinical isolates of this study (Tables 2 and 3, S6 and S7 Figs). Four out of 12 alcohol-based disinfectants (products 8,15–18) had a MIC of 100% and MBC of 75% of the original stock, whereas 6 of the alcohol-based disinfectants (products 2, 3, and 5–7) showed MIC value at 75% and MBC value at 50% of the original stock, for S. *aureus*. Products 1 and 4 had MIC value of 50% and MBC at 25% of the original stock for the same organism.

*E*. *faecalis* were more sensitive than *S*. *aureus* and more tolerant than the other clinical isolates. *E*. *faecalis* presented MIC and MBC values at 75% and 50% of the original stock, respectively, for all the alcohol-based disinfectants (products 1–8, and 15–18).

Lastly, *s*imilar to the reference strains, all the 5 clinical isolates exhibited MIC and MBC values of 12.5–50% and 6.25–25% of the original stock towards the mixed disinfectants (product 9), while the chlorohexidine-based disinfectants (products 11 to 14) showed MIC value at 0.78–1.56% of the original stock without MBC value of the tested serial dilutions (Tables 2 and 3, S3–S7 Figs).

Next, the significance correlation between glycerin concentration and ethanol-antimicrobial efficacy was tested against the 5 clinical isolates using the welch’s t-test. Similar to the previous results, it was found that the antimicrobial effect of the alcoholic-based disinfectant is almost comparable against the tested organisms regardless the glycerin concentration (p > 0.05, Table 4).

## Discussion

Hand sanitizers have been effective in reducing microbial burden when water and soap are not accessible. At the present time, public awareness of using hand disinfectants has been increased in accordance with incidence rate of COVID-19 pathogenic diseases associated with hospitals, communities, or with secondary-infections. That led to the production of numerous hand sanitizers by various companies with the intend of supporting healthcare system and public community in preventing the transmission of post-secondary diseases.

Nevertheless, there were not enough information about laboratory-based efficacy evaluation. This study was conducted with an aim to assess the effectiveness of the common market- based disinfectant products obtained from the local markets in Riyadh, Saudi Arabia against the microbes collected from two different sources (reference strain and clinical isolates). To our knowledge, this is the first study of measuring the efficacy of a large collection of commercial disinfectants in reducing and inhibiting microbial growth in Saudi Arabia.

In this study, the concentration of the 18-tested disinfectants’ active ingredients was evaluated using gas chromatography techniques. As stated earlier that according to the WHO, CDC, and FDA, an alcohol (ethanol or isopropanol) concentration of different forms (gels, sprays, or foams) ranges from 60–95% is considered to be safe and effective in inhibiting microbial organisms [10,33]. Based on GC-FID assay, our results (Table 1) revealed that all the examined alcohol-based disinfectants (products 1–8, and 15–18) have an alcohol concentration of either ethanol or isopropanol falls within the recommended concentration range by the regulatory agencies (60–95% v/v). Product 7 had the lowest detected ethanol concertation (60%); whereas product 16 had the highest detected concentration (92%). This confirms that the tested alcohol-based disinfectants have different ethanol/isopropanol concentrations within the accepted regulatory agencies range. In comparison with previous studies, recent research in 2020 assessed the quality of ethanol-based sanitizers using infrared-spectroscopy where they measured alcohol content [34]. They found that glycerin was one of the most interfering associated contents, where glycerin molecule owns three -OH groups, and it overlapped with ethanol spectrum [34]. This explains that higher ethanol concentration was found in samples which contain glycerin [35]. However, glycerin concentration should not exceed 1.45% as recommended by regulatory agencies, such as the WHO [35].

Based on GC-MS assay, our results (Table 1) revealed that there were eleven products (products 1–8, 15–16, and 18) contained varying percentages of ethanol and glycerin. About 54% (6 /11) of these products contained an equal to or more than 80% alcohol supplemented with more than 1% glycerin (ranges between 1.06–1.55%). Therefore, there was a tendency for manufacturers to increase ethanol concentration when there were more than 1% glycerin in the disinfectant formula.

In this study, the antimicrobial activity of the 18-tested disinfectants was evaluated using standard microbiology MIC and MBC assays (Tables 2 and 3). Each disinfectant was assessed in parallel for activity towards 5 bacterial reference strains and 5 clinical isolates, as the latter would provide more pertinent results from a general health care perspective. The highest the concentration of disinfectants that microorganisms can grow at, the more bacteria can tolerate and survive it.

Broadly speaking, alcohol-based, chlorhexidine gluconate-based, and mixed products were effective in killing the microorganisms of interest (Tables 2 and 3). Overall, our results showed that there was strain-to-strain variation in the effectiveness of some disinfectants. According to recent studies, this variation is potentially due to microbial different genetic makeup due to the acquiring of such mutations which make the bacteria more or less tolerant to biocides, or due to microbial environmental origin [28,36,37]. Our results indicated that microorganism susceptibility towards disinfectants varies and depends on the source where the microbes were collected and the type of the disinfectants.

Although alcohol-based disinfectants have a wide spectrum of activity, our results indicated that they were more active against Gram-negative bacterial strains, where the activity of alcohol-based disinfectants was moderate against Gram-negative bacterial reference strains (*P*. *aeruginosa*, *E*. *coli* and *S*. *enteritidis*), with MIC and MBC values at 25–50% and 25–50%, respectively. In contrast, these alcohol-based products had limited antimicrobial activity against Gram-positive reference bacterial (*S*. *aureus* and *E*. *faecalis*) in which they were inhibited at a very high concentration out of the original stock, which confers their tolerance, where the MIC was 75% and 100%, while the MBC values were 50% and 75%, respectively. The activity against clinical isolates was very similar, where Gram-negative bacteria (*P*. *aeruginosa*, *E*. *coli* and *S*. *enteritidis*) had MIC and MBC values between 25–50% and 25–50%, respectively. In conversely, the Gram-positive bacteria (*S*. *aureus* and *E*. *faecalis*) were inhibited at higher concentration (at 75–100% MIC and 50–75% MBC). The results of this report were similar to the findings of previous studies, where hand sanitizers were more effective in the Gram-negative *P*. *aeruginosa* and *E*. *coli* and less effective in the Gram-positive S. *aureus* [1,2,38,39]. One important mechanism behind this variation is due to bacterial cell wall; while Gram-negative bacteria have a thin peptidoglycan cell wall which can be easily dissolved by alcohols, whereas Gram-positive bacteria acquire a thicker peptidoglycan cell wall that make them less vulnerable towards alcohol products [1,2,38,39].

The emergence of tolerant bacterial strains increases the chances of infections in hospitals. Some potential reasons behind their resistance are due to bad quality of disinfectants (manufacturing and producing) or some genetic mutations in organism’s genome, which make them more tolerant to biocides, or could be due to poor or prolonged storage of these alcoholic products which increases the temperature and causing the evaporation of the main active ingredient, which can easily develop resistance [36]. In accordance with our finding, several reports found that increased MICs of different antiseptics and disinfectants has been shown against MRSA due to the presence of two gene families of chlorohexidine-resistant genes (*qacA/B* and *qacC/D*) and fluoroquinolone efflux transporter protein (*norA*) [40–43]. These gene have been identified in *Staphylococcus* species and mostly found on bacterial plasmids (*qacA/B*) and chromosomes (*norA*) [43]. These genes encode for proton-dependant export proteins and their efflux system appeared to confer resistance to many antimicrobial agents [44]. Another study has demonstrated that multidrug efflux system was possibly contribute to the resistance mechanism of these microbes [45]. In addition, MRSA have an army of toxins and immune avoidance virulence factors for invading host’s hands tissues [46].

The antibacterial activity of chlorohexidine gluconate-based disinfectants (products 11–12) were similar against both reference strains and clinical isolates of both Gram-positive and Gram-negative bacteria. Interestingly, our results were aligned with recent reports where Ekizoglu *et al*. group found that chlorohexidine-gluconate was able in inhibits microbial growth [44]. According to other report, *S*. *aureus* and *E*. *coli* isolates had low MIC values, while *P*. *aeruginosa* and *S*. *maltophilia* isolates had the highest MIC values [43]. Another group showed opposed results, where the microbial inhibition of the Gram-positive *S*. *aureus* by chlorohexidine-gluconate surpassed the Gram-negative *P*. *aeruginosa* [47,48].

Furthermore, the antibacterial activity of mixed- based disinfectants (products 9 and 10) was moderate against all the tested bacteria. Regarding the mixed disinfectants (products 13 and 14), our results revealed that mixing alcohol with low percentage of chlorohexidine showed great antimicrobial effect in all tested organisms, in comparison to product 10 that has higher chlorohexidine content. In accordance with our finding, several studies have shown that mixing alcohol with low percentage chlorhexidine gluconate can increase the efficiency of the disinfectants in killing microorganisms efficiently [49–51]. Taken together, among these tested disinfectants, those containing non-alcoholic chlorohexidine gluconate alone or mixed with other compounds as the principal active ingredient seemed to offer the greatest antibacterial activity across all tested bacterial species than the alcohol-based disinfectants, regardless of the source of origin.

Few literature studies evaluated the relationship between glycerin concentration and antibacterial efficacy for ethanol-based disinfectants. According to previous studies, it was observed that high glycerin levels (i.e., exceeding the 1.45% of glycerin) could reduce the antimicrobial efficacy of the alcohols in the microbiology laboratory [52,53]. Conversely, the same group found that reducing glycerin levels led to a stronger reduction in skin microbial flora would lead to skin irritation [52–54]. Thus, the minimum required concentration of glycerin remains unknown and according to the literature, the concentration of glycerine varies according to the climates [52,53].

In the current study, it was noticed that only 2 out of the 11 ethanol-based disinfectants with the lowest glycerin content (products 1 and 8 with glycerin levels of 0.32 and 0.41%, respectively) demonstrated similar bacterial inhibition against reference and clinical *E*. *faecalis* isolate compared to other alcohol-based products with high glycerin content (e.g., products 2 and 16). Our study also showed a very interesting finding, where it seems that the variation in both glycerine and ethanol levels did not significantly alter their corresponding bacterial inhibition in all the tested alcohol-based disinfectants (*p* > 0.05, Table 4). For example, the alcohol-based product 7 (60% ethanol alcohol and 1.07% glycerin) and product 16 (92% ethanol alcohol and 1.66% glycerin) showed similar inhibition and bactericidal effects against all the 5 reference strains and 3 of the clinical isolates (*P*. *aeruginosa*, *E*. *coli*, and *E*. *faecalis*) despite their variance ethanol levels (Tables 2 and 3). Therefore, this study showed that there was no significant correlation between the MIC/MBC values and the increase or the decrease in the concentration of glycerin of all tested products (*p* > 0.05, Table 4). In accordance with this observation, a recent randomized, double-blinded, cross-over Brazilian study examined the effect of different ethanol-based hand rub disinfectants contained a variety of glycerin concentration on 40 health-care workers; and they found that the 0.5% glycerin led to better ratings of skin tolerance than the other products and therefore, may offer better balance between antimicrobial activity and skin tolerance [53]. Obviously, this observation is not conclusive owing to the limited number of tested products, yet it might support the previous studies. Future studies with larger sample size may provide a better understanding of this perspective.

Lastly, our overall findings indicated that the tested disinfectant products of the local Saudi market followed the guidelines and regulation of the Saudi Food and Drug Authority (SFDA); where the SFDA recommends the pharmacopeial specifications for ethanol and isopropyl alcohol which is according to the European Pharmacopoeia (Ph.Eur), the United States Pharmacopeia (USP) and international pharmacopoeia. These specifications are critical quality standards that are proposed and justified by the manufacturer and approved by SFDA as conditions of approval. However, due to COVID-19 pandemic, SFDA has established minimum requirements to help local manufacturers with the purpose of increasing the production of ethanol and isopropyl alcohol as active ingredients that applied for hand sanitizers. The manufacturers should provide a certificate of analysis that includes the name of the company, name of the product, the active ingredients, the batch number, the release and expiry date, a description of the alcohol examination method(s) used, limits of the test, and actual results of the tests and dated the signature by the authorised personnel. A full detailed of the SFDA regulations in approving disinfectants manufacturing are found in (www.sfda.gov.sa).

The present study is subject to some limitations, where only bacterial isolates were tested not viral strains due to the laboratory setup constrain for culturing the viruses. In addition, the active ingredients of all the disinfectant products were evaluated using gas-chromatography assay except the products that contain the mixture of chlorhexidine/hexamidine/chlorocresol in their label due to the unavailability of the standard reagents for these compounds (Table 2, products 9, 11, and 12). Based on our study, some recommendations need to be considered. It is necessary to reduce the concentration of active ingredient chlorhexidine gluconate in the disinfectant-manufacturing, as the results of this report showed that two chlorohexidine-based disinfectants (products 11 and 12) which contain 2% and 4% of chlorhexidine gluconate, respectively, were diluted several times (7x dilutions) and their effect were excellent in killing bacteria. Therefore, the concentration levels for these non-alcoholic-based (chlorohexidine gluconate) products needs to be re-evaluated, to use the right concentration and right amount of disinfectants, which can kill the pathogenic bacteria without leading to genetic mutation, which can cause bacterial resistance to chlorhexidine gluconate. Therefore, it is crucial to save the chlorohexidine high concentration for future use, when is needed. The regulatory authorities in Saudi Arabia (SFDA) shall keep enforce their strict quality control measures during products production, distribution and routine laboratory inspections to ensure the efficacy of the commercial disinfectants in the Saudi market and to implement regular assessment of disinfectants efficacy in killing bacteria and viruses especially the dominant microbial strains in Saudi Arabian hospitals in order to make sure that the used disinfectants are helping in preventing the transmission of infectious diseases.

## Conclusion

The use of disinfectants is important in preventing the transmission of infectious diseases in hospitals and between patients as well as in the community. Overall, our results showed that the tested disinfectant products of the local Saudi market are effective in inhibiting the growth of the selected bacterial strains, which indicated that these products were under a good surveillance by the local authorities. Alcohol-based disinfectants efficiently repressed bacterial growth; but, the rate of the inhibition of these products was lower than the chlorohexidine-alcohol-based disinfectants. However, alcohol-based products were effective in reducing bacterial growth. In addition, this study showed that there was no significance correlation between the concentration of glycerin and its effect on the antimicrobial efficacy of alcohol-based products against pathogenic bacteria. It is necessary to establish a regular surveillance program for assessing the effectiveness of the available disinfectants in the market of Saudi Arabia to ensure having high quality disinfectants for hospitals and the community. It is also important to study the molecular mechanism of resistance towards disinfectants in order to understand how microbes evolve overtime; hence to find solutions for supressing or overcome any emerged resistance. In addition, further studies should be conducted to document and to confirm the negative correlation between the concentration of glycerin and its effect on the antimicrobial efficacy of alcohol-based products against multiple pathogenic bacteria *in vitro* and *in vivo* taking into account product formulation and effective concentration.

## Supporting information

S1 Fig This table demonstrates the mean optical density (OD) ± standard deviation (SD) which reflects the MIC % of the 3 independent biological replicates. (TIF)

S2 Fig This table demonstrates the mean OD ± SD which reflects the MBC % of the 3 independent biological replicates. (TIF)

S3 Fig A representative bar graph demonstrated the efficiency of all disinfectant products in inhibiting the growth of *P*. *aeruginosa* (A) ATCC reference strain and (B) clinical isolates.
(TIF)

S4 Fig A representative bar graph demonstrated the efficiency of all disinfectant products in inhibiting the growth of *E*. *coli* (A) ATCC reference strain and (B) clinical isolates.
(TIF)

S5 Fig A representative bar graph demonstrated the efficiency of all disinfectant products in inhibiting the growth of *S*. *enteritidis* (A) ATCC reference strain and (B) clinical isolates.
(TIF)

S6 Fig A representative bar graph demonstrated the efficiency of all disinfectant products in inhibiting the growth of *S*. *aureus* (A) ATCC reference strain and (B) clinical isolates.
(TIF)

S7 Fig A representative bar graph demonstrated the efficiency of all disinfectant products in inhibiting the growth of *E*. *faecalis* (A) ATCC reference strain and (B) clinical isolates.
(TIF)

S1 Data (XLSX)

## Abbreviations

ABHR: Alcohol-based hand rub
CDC: the center for disease control and prevention
COVID-19: coronavirus disease 2019
FDA: food and drug administration
GC-FID: GC- flame ionization detector
GC-MS: gas chromatography-mass spectrometry
KIMARC: king Abdullah international medical research center
MALDI-TOF MS: Matrix-Assisted Laser Desorption/Ionization-Time of Flight mass spectrometry, bactericidal concentration
MBC: minimum bactericidal concentration
MIC: minimum inhibitory concentration
MRSA: methicillin-resistant Staphylococcus aureus
Ph.Eur: European Pharmacopoeia
SARS-Cov: severe acute respiratory syndrome coronavirus
SARS-Cov-2: severe acute respiratory syndrome coronavirus 2
SFDA: Saudi food and drug authority
TSA: tryptic soy agar
TSB: double concentrated tryptic toy broth
TSD: tryptone saline diluent
USP: the United States Pharmacopeia
WHO: world health organization
