# Supplementary figures and images for: Assessment of disinfectant efficacy in reducing microbial growth
Source: PLoS One. 2022 Jun 27;17(6):e0269850. doi: 10.1371/journal.pone.0269850 (PMC9236243; doi:10.1371/journal.pone.0269850)

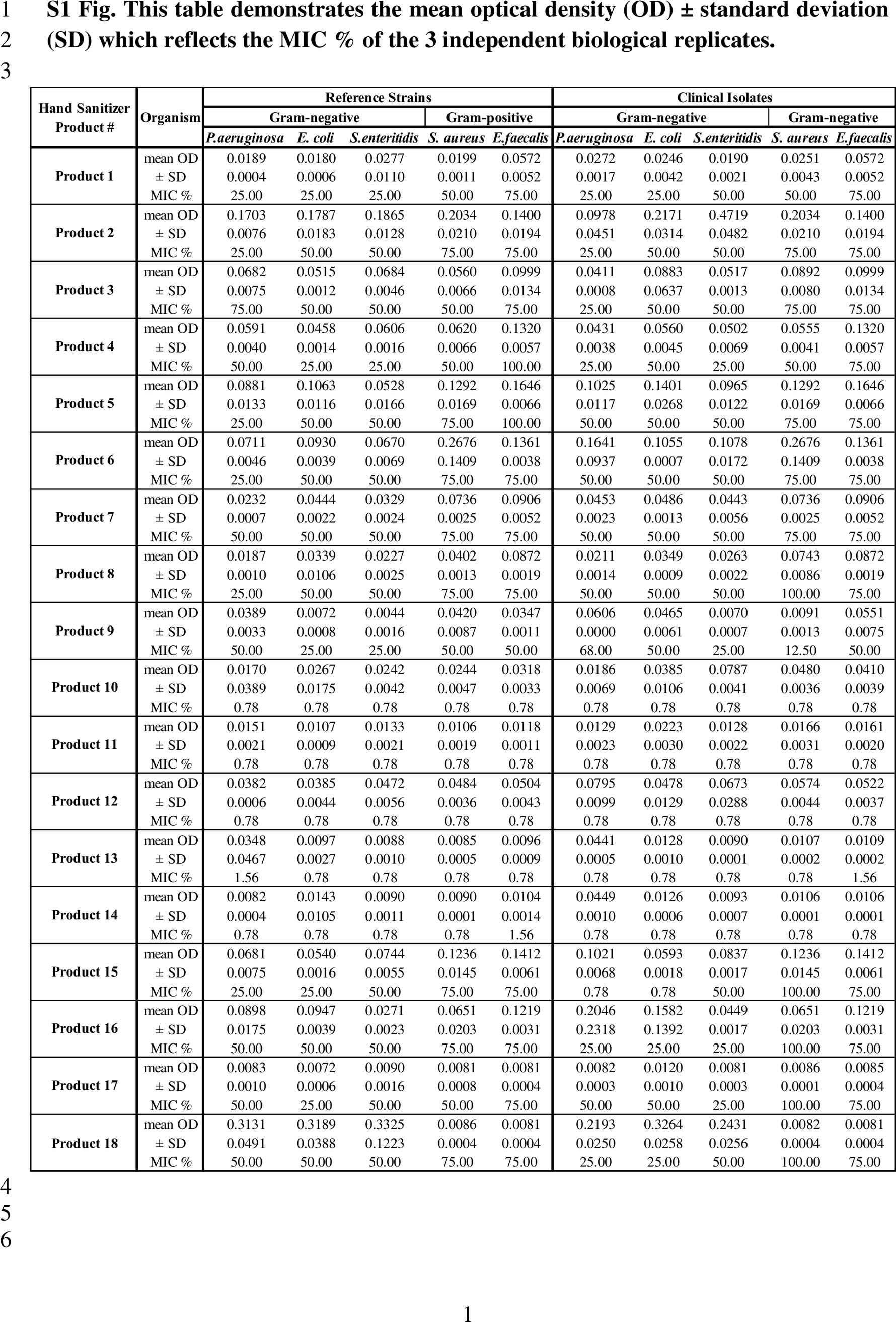

Supplement: S1 Fig — (TIF) [file pone.0269850.s001.tif]

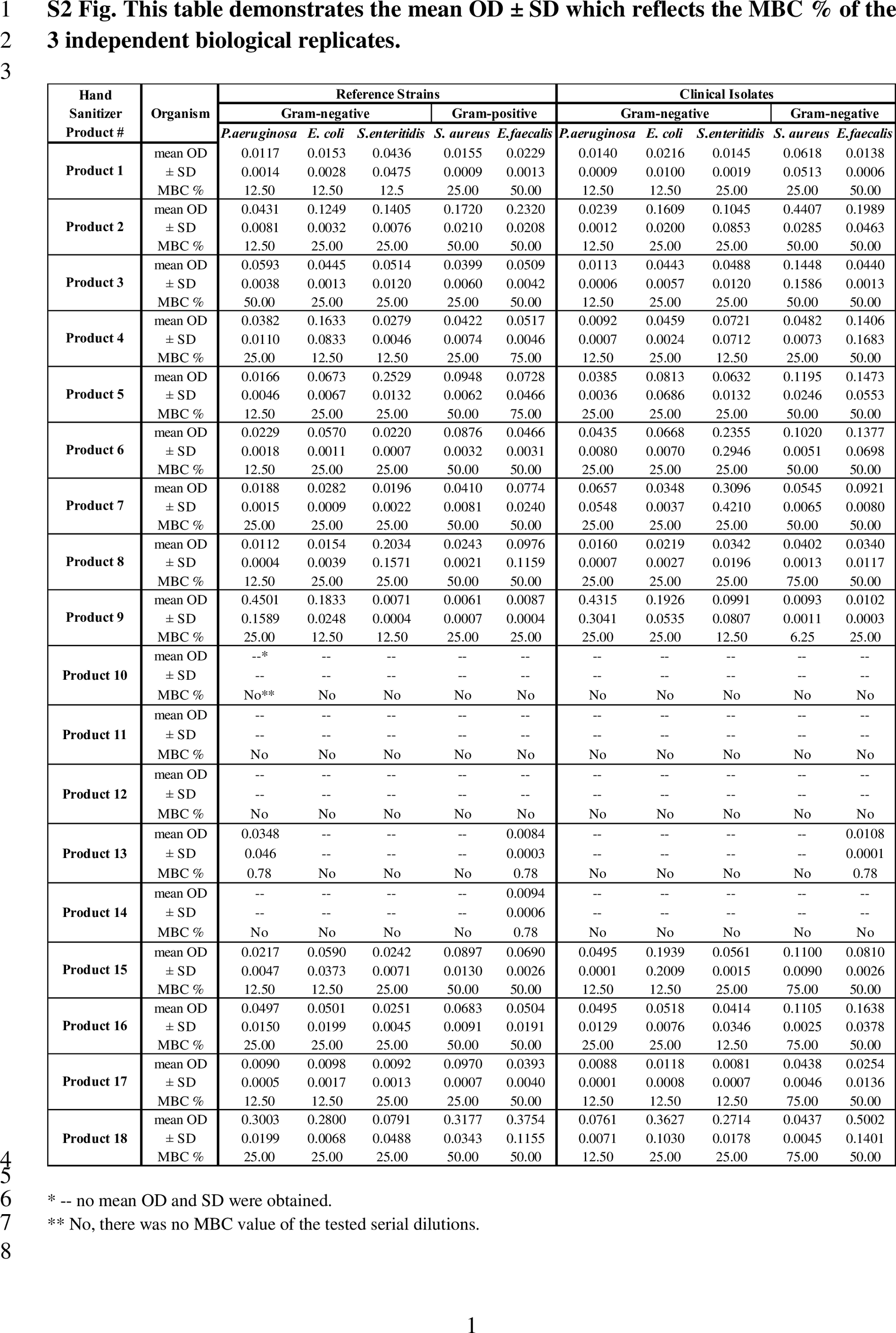

Supplement: S2 Fig — (TIF) [file pone.0269850.s002.tif]

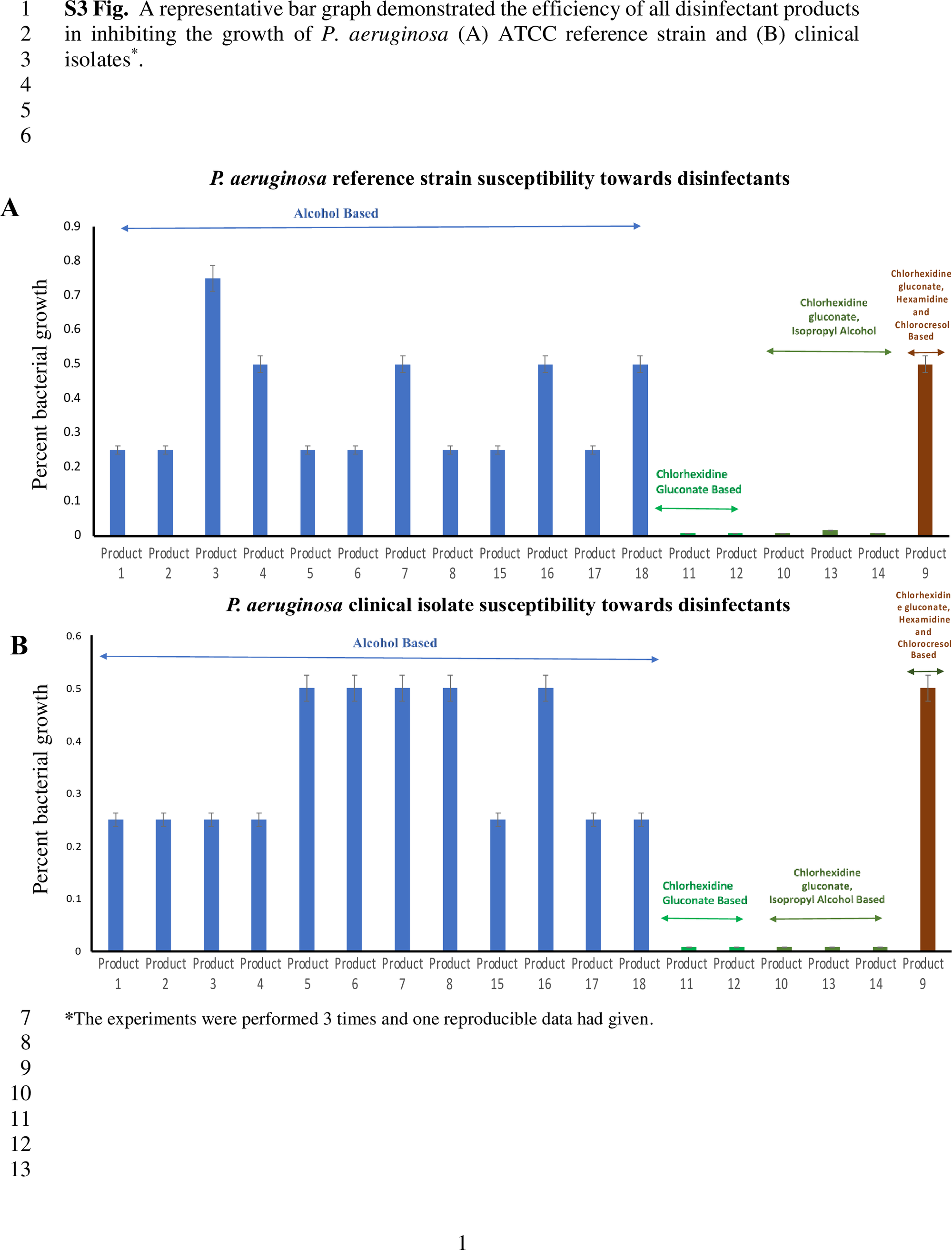

Supplement: S3 Fig — A representative bar graph demonstrated the efficiency of all disinfectant products in inhibiting the growth of P. aeruginosa (A) ATCC reference strain and (B) clinical isolates. (TIF) [file pone.0269850.s003.tif]

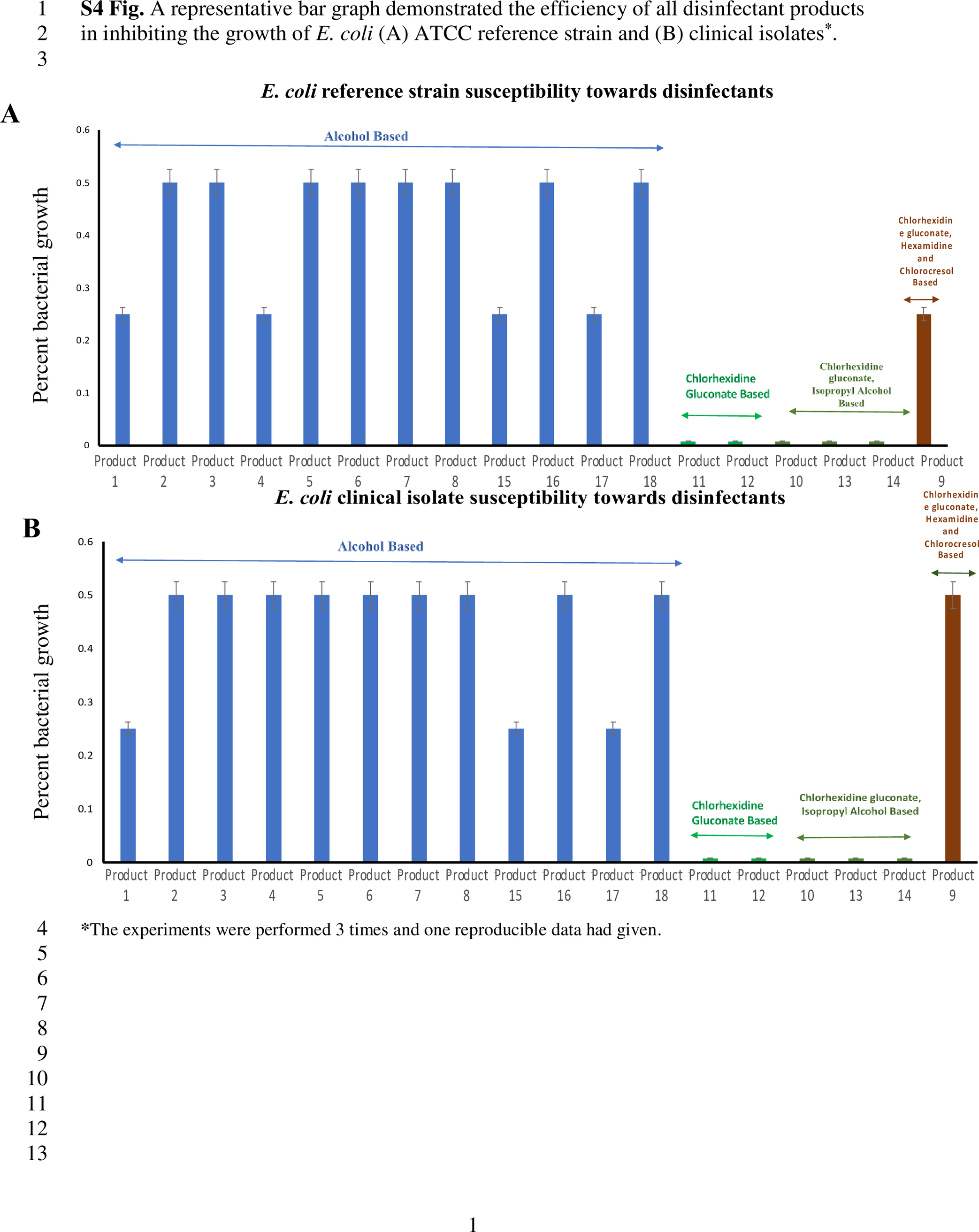

Supplement: S4 Fig — A representative bar graph demonstrated the efficiency of all disinfectant products in inhibiting the growth of E. coli (A) ATCC reference strain and (B) clinical isolates. (TIF) [file pone.0269850.s004.tif]

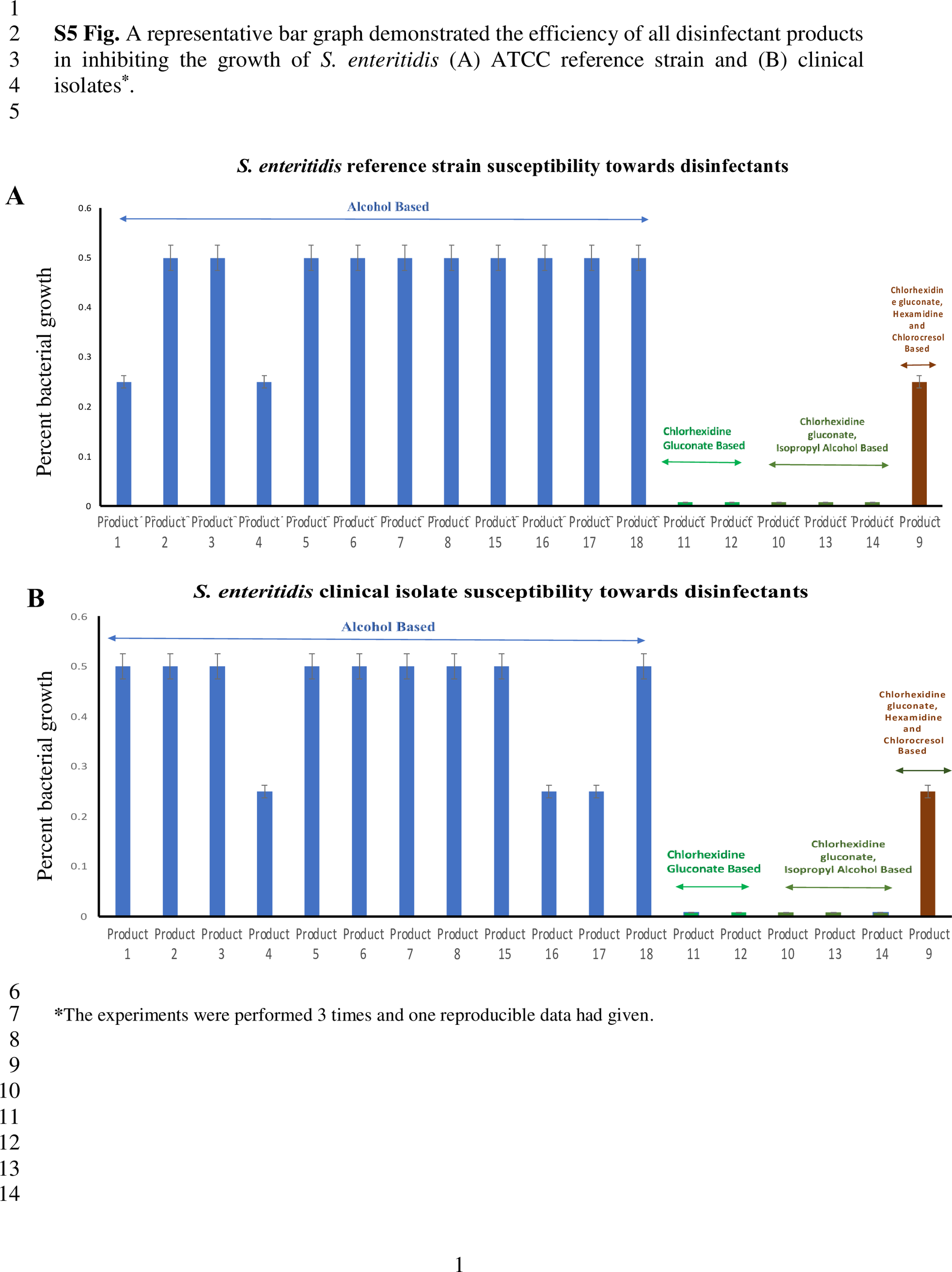

Supplement: S5 Fig — A representative bar graph demonstrated the efficiency of all disinfectant products in inhibiting the growth of S. enteritidis (A) ATCC reference strain and (B) clinical isolates. (TIF) [file pone.0269850.s005.tif]

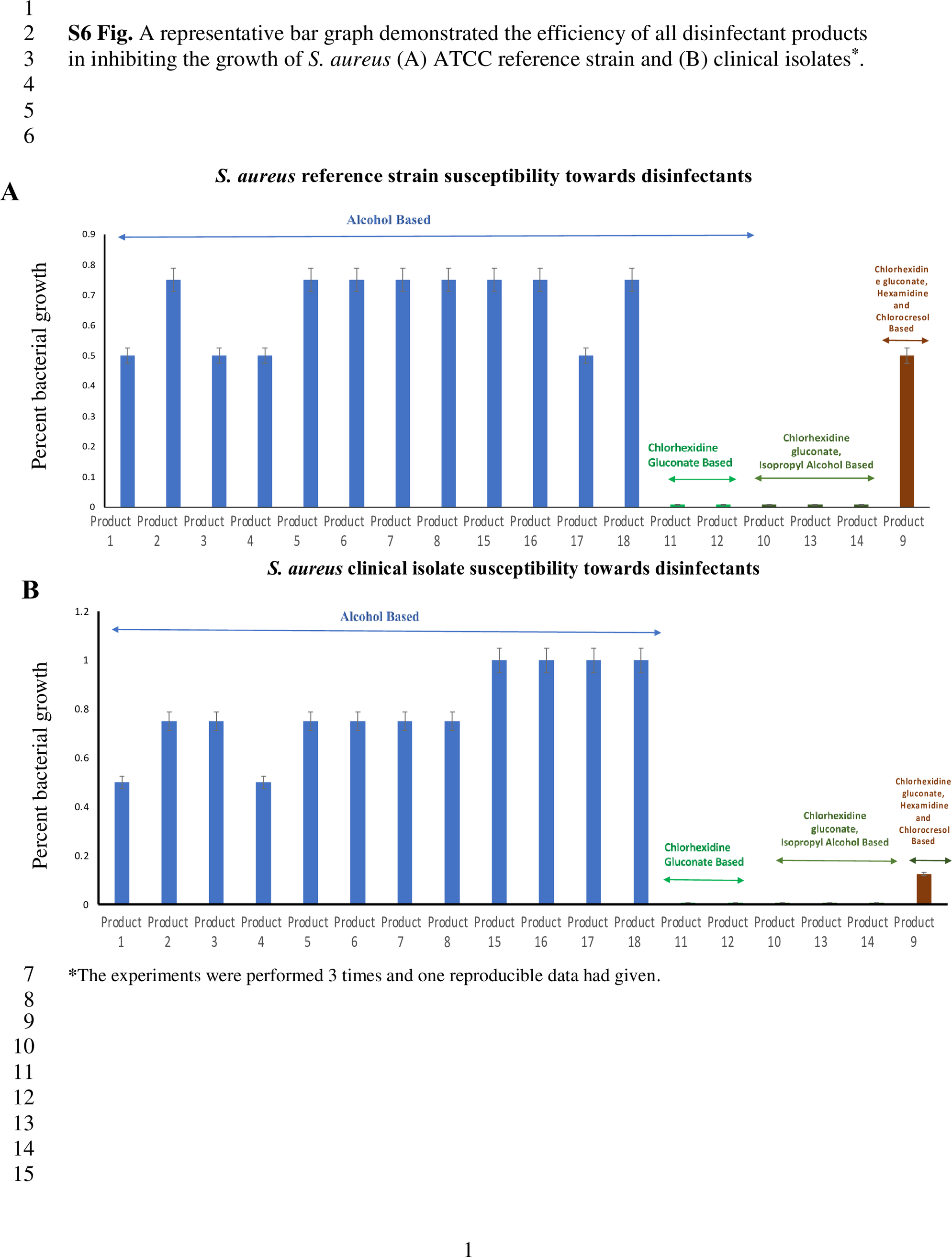

Supplement: S6 Fig — A representative bar graph demonstrated the efficiency of all disinfectant products in inhibiting the growth of S. aureus (A) ATCC reference strain and (B) clinical isolates. (TIF) [file pone.0269850.s006.tif]

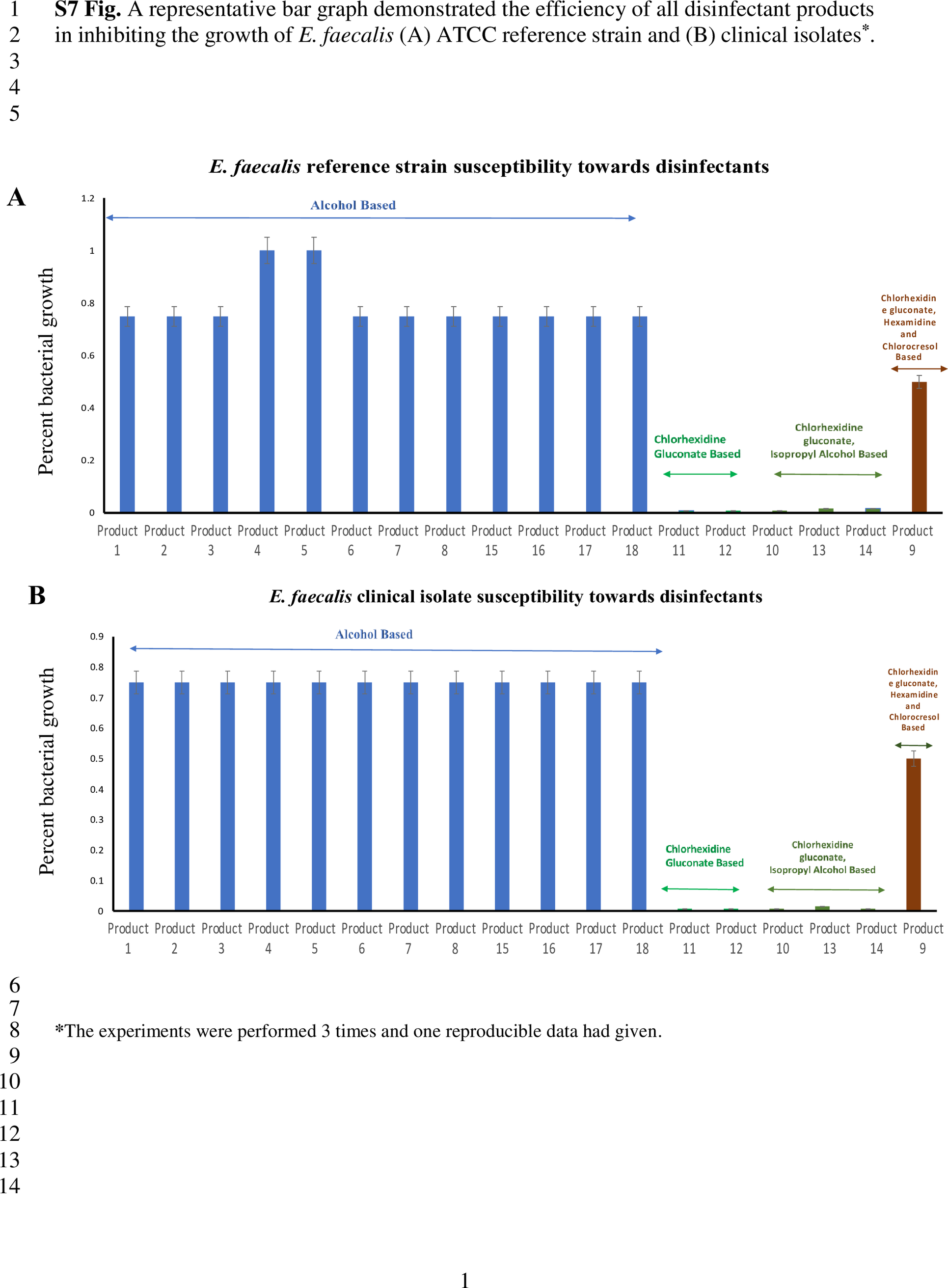

Supplement: S7 Fig — A representative bar graph demonstrated the efficiency of all disinfectant products in inhibiting the growth of E. faecalis (A) ATCC reference strain and (B) clinical isolates. (TIF) [file pone.0269850.s007.tif]
